# On a Case of Epilepsy Treated by Tracheotomy

**Published:** 1859-04

**Authors:** Edward Jackson Riccard


*On a Case of Epilepsy Treated by Tracheotomy.*—By Edward Jackson Riccard, M. D.

Anne V——, aged thirty, single, has been subject to fits for the last seven years; for several months she has had two or three fits in a day, caused, it is supposed, by the neglect of her parents. She is one of four children; one of whom died in infancy, and the other three are all living. Her mother died of dropsy, aged fifty-nine years; her father is still living, and a hale man of sixty-seven years of age. Previous to having a fit she feels "lost in her head." She has a vacant look, and her intellect has been decidedly suffering for the last two or three years. She has always had sedentary employment.

On the 5th of October, 1856, at eleven, A. M., assisted by my pupil, Mr. Waterworth, I performed the operation of tracheotomy, my patient being under the influence of chloroform. The skin and integuments being first divided, the beak of the instrument (the most perfect of its kind, made by Messrs. Weiss & Co.,) was introduced through the trachea, between the first and second rings, and traction steadily persevered in until the opening was large enough to admit the tracheal tube to be passed into it. Almost instantaneously hæmoptysis occurred to an alarming extent, and continued for several minutes; it then got gradually less, and within half an hour had almost entirely ceased, but not before a considerable quantity of blood was lost both *through the tube* and the mouth. The tube was fastened by an elastic band round the neck, with a pad on either side, to prevent pressure on the jugular vein. At one o'clock, P. M. she was quiet, pulse 76, and no blood was expectorated. Five o'clock; has twice taken tea, and is tolerably comfortable. At seven o'clock she was left by her nurse for a few minutes, who, on her return, found her on the floor in a fit, which lasted only about a minute. The hæmoptysis returned slightly. Pulse 80. Nine o'clock; has been vomiting; no return of the hæmoptysis; complains of thirst; pulse 78. She was ordered tincture of digitalis, one drachm; tincture of hyoscyamus, one drachm and a half; antimony wine, three drachms; the liquor of acetate of ammonia, an ounce and a half; water sufficient to make six ounces; mix. Two tablespoonfuls to be taken every six hours.

Oct. 6th.—Nine o'clock, A. M: Passed a comfortable night; had a slight fit about four o'clock this morning, but it did not last more than two minutes; had no cough; took a cup of tea with a biscuit soaked in it; no disphagia; pulse 80; passed urine twice in the night; does not complain of thirst.

Ten P. M.: Is tolerably easy; had no cough; passed urine three or four times; has taken tea with cake soaked in it several times; complains of difficulty in swallowing; bowels have not been moved.

7th.—Eleven A. M.: Passed a comfortable night; had no fit; feels the hot breath come through the tube; had no cough; no thirst;—"has not felt so free from fits for years;" pulse 82; tongue clean. Continue the mixture. Eight P. M.: Inclined to sleep; the breath could be slightly felt coming through the tube; bowels moved once; pulse 80.

8th.—Eleven A. M.: Passed a very good night; had no fit or "sensation" in the head; expresses herself very grateful for the relief she has received from the operation; pulse 72; tongue clean; feels the breath blow on her chin from the tube whilst drinking a cup of tea.

9th.—Still progressing very favorably; is down stairs; has had no fit; pulse 73; secretions natural; tongue clean.

10th.—Has had a good night; once through the night she felt a slight twitching of the lips, which used to be a premonitory symptom of a fit, but she had no fit; pulse 72; a rather copious discharge of mucous through the tube.

11th.—Still progressing favorably; a thick, healthy discharge from the wound in the neck; pulse 82.

12th.—Has had a good night; for a few minutes after she got down stairs this morning, she felt as if she were going to have a fit; this feeling soon passed off. Took out the tube and cleaned it.

17th.—Exeessive granulations appearing through the posterior opening of the tube, caustic was applied; has had no fit, and is improving.

18th.—Removed the tube, and introduced a double one having only one opening.

19th.—Still going on well; took out the tube and showed her how to clean it, and introduced it again without any difficulty. She returned into the country to-day.

Dec. 13th.—Met the patient to-day. She has still slight fits or "sensations," but they do not affect her as they did before she was operated on; "they do not throw her down;" says "she would not have the operation undone for all the world."

The patient continued free from fits until the first week in May of the following year. She then applied to me again, having had some severe fits, and several attacks of hæmoptysis. On examining her neck, I found the point of the tracheal tube external and anterior to the trachea, just held beneath the skin and integuments only. She had allowed the elastic band to get too slack, and "Nature's own process" had gradually removed it from its position. She returned again to the country that evening, and had two fits on her way home. The next day I was sent for, and, on my arrival, she had a very severe fit, which lasted nearly an hour; and during the whole of that time she continued expectorating blood. She lingered on in this state until the 31st of May, when she died, having been quite insensible since the 17th.

The termination of this case was unfortunate; but the progress of it throughout speaks much in favor of the operation as a means of mitigating the severity of so serious a disease as epilepsy. The *great suggester* of the remedy well deserves, as he has, the thanks of the world. His fame is, indeed, world-wide.—*London Lancet.*

